# Selective vulnerability of motoneuron and perturbed mitochondrial calcium homeostasis in amyotrophic lateral sclerosis: implications for motoneurons specific calcium dysregulation

**DOI:** 10.1186/2052-8426-2-26

**Published:** 2014-08-14

**Authors:** Manoj Kumar Jaiswal

**Affiliations:** Center for Neuroscience and Regenerative Medicine, 4301 Jones Bridge Road, 20814 Bethesda, MD USA; Department of Anatomy, Physiology and Genetics, School of Medicine, USUHS, 4301 Jones Bridge Road, 20814 Bethesda, MD USA

**Keywords:** Amyotrophic lateral sclerosis (ALS), Motoneuron, Calcium dysregulation, Mitochondria, ER-mitochondria calcium cycle (ERMCC), Selective vulnerability, Calcium buffering, Multifactorial disease, Multidrug therapy

## Abstract

Amyotrophic lateral sclerosis (ALS) is a lethal neurodegenerative disorder characterized by the selective degeneration of defined subgroups of motoneuron in the brainstem, spinal cord and motor cortex with signature hallmarks of mitochondrial Ca^2+^ overload, free radical damage, excitotoxicity and impaired axonal transport. Although intracellular disruptions of cytosolic and mitochondrial calcium, and in particular low cytosolic calcium ([Ca^2+^]_c_) buffering and a strong interaction between metabolic mechanisms and [Ca^2+^]_i_ have been identified predominantly in motoneuron impairment, the causes of these disruptions are unknown. The existing evidence suggests that the mutant superoxide dismutase1 (mtSOD1)-mediated toxicity in ALS acts through mitochondria, and that alteration in cytosolic and mitochondria-ER microdomain calcium accumulation are critical to the neurodegenerative process. Furthermore, chronic excitotoxcity mediated by Ca^2+^-permeable AMPA and NMDA receptors seems to initiate vicious cycle of intracellular calcium dysregulation which leads to toxic Ca^2+^ overload and thereby selective neurodegeneration. Recent advancement in the experimental analysis of calcium signals with high spatiotemporal precision has allowed investigations of calcium regulation *in-vivo* and *in-vitro* in different cell types, in particular selectively vulnerable/resistant cell types in different animal models of this motoneuron disease. This review provides an overview of latest advances in this field, and focuses on details of what has been learned about disrupted Ca^2+^ homeostasis and mitochondrial degeneration. It further emphasizes the critical role of mitochondria in preventing apoptosis by acting as a Ca^2+^ buffers, especially in motoneurons, in pathophysiological conditions such as ALS.

## Introduction

Amyotrophic lateral sclerosis (ALS) is an incurable, adult-onset, deadly neurodegenerative disorder distinguished by the progressive degeneration of a defined motoneuron (MN) population in the brain stem, spinal cord and motor cortex. ALS leads to paralysis, atrophy and death within 5 years [1]. Currently, there are no effective drug formulations for cures and riluzole is the only FDA approved treatment available for this devastating disease. The majority of ALS cases are sporadic, but ~10% of ALS cases are familial ALS (fALS). About 20% of these familial cases are caused by dominantly inherited mutations in the gene encoding the enzyme Cu/Zn-superoxide dismutase (SOD1) and are the most common [2]. Mutations in a number of other genes also cause familial ALS; including mutations in autosomal dominant familial MN disease include fALS types 3 [3], 5 [4], 6 or FUS gene [5, 6], 7 [7], 8 [8], 9 or ANG gene [9], 10 or TDP-43 (TARDBP gene) [10–12], 11 or Figure four gene [13], NF-H gene [14], DAO gene [15], X-linked [16], C90RF72 [17–19], alsin [20] and MND with FTD [21].

Unfortunately, the discovery of these mutant genes has not yet advanced into useful ALS model organisms, allowing most work described in literature have been conducted on mutant SOD1 cells, rodents or on patients (both familial and sporadic). Although the process of MN degeneration both in sporadic and familial forms is still little understood, it is generally agreed that there are cell-specific features, particularly impaired uptake of Ca^2+^ into mitochondria and low content of Ca^2+^ binding proteins which modulate physiological as well as pathophysiological processes and may render MNs selectively vulnerable to degeneration [22–25]. In both SOD1-mediated and sporadic sALS, reports demonstrate that mitochondrial alterations, including morphological changes, enhanced activity of complexes I, III, and IV, and increased reactive oxygen species (ROS) generation, are key factors in its pathogenesis [8, 26–30]. These factors are further exacerbated by observations indicating that the impaired spinal cord and vulnerable spinal cord neurons trigger the functional decline of MNs in neighboring regions, leading to the onset of pathology in ALS [31–34]. The breakdown of mitochondrial membrane potential (ΔΨm), excitotoxic stimulation of AMPA/kainite receptors and age associated MN damage reported by several groups may also be a factor to ALS pathogenesis [35–38].

Impaired intracellular Ca^2+^ homeostasis, rather than direct mitochondrial disruption, is supported by observations that neurons lost early in the disease progression have intrinsically poor cytoplasmic Ca^2+^ buffering capabilities, due to the absence of Ca^2+^ binding proteins such as calbindin D_28K_ (CB-D_28K_) and parvalbumin (PV) [22, 36]. These findings are in agreement with observations that low cytosolic Ca^2+^ buffering ability acts as a primary risk factor for MN deterioration, while increases in cytosolic Ca^2+^ buffering capacity protects vulnerable MNs from degeneration, both *in-vitro* and *in-vivo* [25, 36, 39, 40]. In addition to the mechanisms of Ca^2+^ toxicity present in most cell types, there are several other characteristics of neurons that make them especially vulnerable. Disturbances of glutamate-mediated neurotransmission and the subsequent glutamate release triggered by Ca^2+^ entry [41] increase extracellular glutamate levels leading to excitotoxicity. This problem is further compounded by reduced glial glutamate uptake caused by oxidative damage to excitatory amino acid transporter 2 (EAAT2) [42]. Studies of fALS in cell lines and in various mouse models that induce Ca^2+^ disturbance via the inhibition of glial glutamate transport by mtSOD1 yield phenotypes similar to effects proposed in sALS [43]. In addition, in cell culture experiments, partial protection was also obtained by treatment with the Ca^2+^channel-blocker nifedipine, implicating Ca^2+^ entry through voltage-gated Ca^2+^ channels in mediating the toxicity of mtSOD1^G93A^ in MNs [44]. In addition to damaging the cell of origin, ROS generated in MNs can cross the plasma membrane and damage glutamate transporters in neighboring astrocytes [41, 45]. Further evidence also indicates that the patients with minor SOD1 mutations had the shortest disease durations. In other words, an accelerated disease course is found for the mutants that are more functionally impaired. Since hydrogen peroxide plays an essential role in the formation of SOD1 monomers, dissociation of the dimeric SOD1 molecule monomers should be as essential important process for APS pathogenesis [46].

However, in ALS pathology, the cause of dysfunction has become highly contentious after latest discoveries where ALS astrocytes were shown to secrete substances that are selectively toxic to MNs [47, 48]. This means that multiple cell types are involved in the disease process. By adding or deleting mtSOD1 in specific cell types e.g. astrocytes [49, 50], microglia [51], schwann cells [52], motor neurons [53] and fibroblast [54] it is possible to influence the disease. Nonetheless, MNs are the cells that directly cause the loss of muscle and limb movement. It is shown that mtSOD1 expressed solely in MNs is sufficient to kick off the disease, although disease progression is slow compared to ubiquitous expression of mtSOD1 [53]. In patients, MNs in the motor cortex, HMNs and FMNs of brain stem and spinal cord undergo cell death selectively. There are a number of hypothesis that explain cell type selectivity, including the extraordinary long axons, the large soma and the poor intracellular calcium buffering capacity and excitotoxicity. It is the latter hypothesis that shall be elaborated on in this review, the only mechanism proved to play a role in patients. Calcium, which causes neuronal death in excitotoxicity, can originate from either the extracellular space or from intracellular stores. In general resting calcium in the extracellular space is ~ 3–4 orders of magnitude higher than in the intracellular stores [55]. As reported previously, MNs are excited by glutamate from the pre-synaptic neuron that binds to glutamate receptors on the postsynaptic MN, which are, among others, the AMPA receptors (AMPA_R_). The AMPA_R_ found on MNs are mainly calcium permeable in vitro and in-vivo [56–59], which may explain the selective vulnerability of MNs to excitotoxic cell death. In addition, extracellular calcium entry via these calcium permeable AMPA_R_ is responsible for selective MN death, as MN death is inhibited by selective blockers of calcium permeable AMPA_R_ [57, 58]. Also, electrophysiological experiments showed that the AMPA_R_ receptors of MNs had a lower rectification index and a higher relative calcium permeability ratio than other neurons [59]. In conclusion, MNs express calcium permeable AMPA_R_ receptors, which could partially explain their pronounced and selective vulnerability to excitotoxic insults [59]. Further explanation for selective MN vulnerability in this cell type has to deal with the increased amount of intracellular calcium. Studies into the calcium homeostasis in MNs have shown a diminished calcium buffering capacity distinguishing ALS-vulnerable from resistant MN types. To begin with, ALS-vulnerable spinal and brain stem MNs in mice display a low endogenous calcium buffering capacity as demonstrated by patch clamp and microfluorometirc calcium measurements [60, 61]. In addition, ALS-resistant oculomotor neurons contain a larger calcium buffering capacity than ALS-vulnerable MNs, as measured by similar microfluorometirc calcium measurements [62].

The kinetics of calcium signals in MNs are shaped by multiple mechanisms including Ca^2+^ influx, Ca^2+^ uptake and release phenomenon, MN specific Ca^2+^ buffering and extrusion across cellular membrane and microdomains in vicinity [63, 64]. Over the past decade, there has been an increased understanding of local communications between Ca^2+^ microdomains and its upstream and downstream target signaling pathways. In particular, an impaired interaction between calcium signaling and mitochondrial processes has been identified as one cellular factor contributing to neurodegenerative processes like those found in motoneuron diseases. In addition, previous studies also indicate the importance of calcium and mitochondria for normal physiological function of MNs [62, 65]. Under pathophysiological conditions, low [Ca^2+^]i buffering and a robust interaction between metabolic processess and [Ca^2+^]i have been related with selective and severe MN damage resulting from excitotoxic stress and disruptions of cellular and mitochondrial Ca^2+^ homeostasis [66, 67]. Considering the prime participation of mitochondria not only in calcium homeostasis directly but also in the energy transduction (to operate other Ca^2+^ clearing mechanisms) and in enacting apoptosis, this aspect of cellular function is of immense importance.

The influence of mitochondrial dysfunction on intracellular Ca^2+^ homeostasis and its role in MN death are fascinating issues that warrant in depth debate. Despite rigorous research, since description of ALS by Charcot ~ 130 years ago, the cellular and molecular abnormalities, which lead to loss of specific MNs, are still dodging the scientific community. Obviously, unraveling the connecting association between mitochondrial dysfunction, calcium dysregulation, and neuronal demise is vital for the understanding of ALS pathogenesis. The aim of this review is to discuss and determine the role of mtSOD1 toxicity in cellular Ca^2+^ homeostasis and mitochondrial dysfunction in MNs of ALS. This review will focus on what has been learned about motoneuron specific calcium dysregulation and perturbed cellular calcium homestasis in ALS from genetically modified animals and cell culture models. Taken together, this review proposes an intregative view, describing mechanisms and critical elements of the pathology of mtSOD1-mediated motoneuron degeneration in ALS.

## Review

### Disease mechanism in amyotrophic lateral sclerosis

Many pathophysiological mechanisms have been suggested to play a role in the etiology of ALS. Corresponding to clinical features, ALS is characterized by a progressive loss of spinal, brains stem and cortical MNs. MN damage as a result of oxidative stress and excitotoxicity is one of the key hypothesis in ALS etiology. The present evidence also supports the hypothesis of mitochondrial dysfunction acting with oxidative stress to cause neurodegeneration via apoptotic mechanisms. Oxidative stress is also linked with other suggested disease mechanisms such as excitotoxicity causing an increase in [Ca^2+^]i, which subsequentially leads to increased nitric oxide formation. Peroxynitrite, produced by the reaction of superoxide anions and nitric oxide, can consequently lead to oxidative disruption [42]. Glutamate excitotoxicity is another mechanism implicated in ALS pathogenesis through disruption of [Ca^2+^]i homeostasis and free radical production. For example, in human ALS (hALS), overexpression of glutamatergic synapses leading to excessive Ca^2+^ influx has been linked with MN degeneration [68]. The oxidative stress manifested in ALS might also prop up increased excitotoxicity, as glutamate transporters are predominantly susceptible to disruption by oxidants, and oxidative modifications to the transporters have been reported in ALS and the mtSOD1 mouse model [69]. The roles of individual cellular domains at the organelle level suggest that high calcium buffering enhances MNs vulnerability [70, 71]. In summary, ALS comprises the interplay of numerous mechanisms from initiation and spread of MN cell death by mitochondrial dysfunction and/or by enhanced MN excitability by intracellular calcium overload. Therefore the etiology of the disease is most likely to be multifactorial [72, 73]. This article will focus on and further discuss the hypothesis and key mechanism that have been most influential in the present and past decade of ALS research.

### Oxidative stress sensitivity and mitochondrial dysfunction

Oxidative stress occurs from an imbalance between the production of ROS and the ability of the system to remove ROS or repair the damage caused because of it, and to reinstate the existing reducing ambiance [68]. Oxidative stress and mitochondrial dysfunction are implicated in the pathogenesis of both normal aging and neurodegenerative diseases. There is ongoing debate as to whether oxidative stress is a primary cause of degeneration or it is merely an end result of some other toxic insult. Although oxidative stress is implausible to be the solitary cause of disease initiation, in several cases it may be enhance vulnerability of homeostatic control mechanisms from handling with a toxic insult into a vicious cycle of cellular insults that results in MN degeneration. MN damage as a consequence of oxidative stress is supposed to be the key premise in ALS. A number of studies have established the existence of elevated oxidative metabolism in ALS, for example finding of increased biochemical markers of oxidative injury in post-mortem samples from ALS patients [38]. Free radical scavenging proteins like SOD1, mitochondrial manganese SOD (SOD2), catalase, and cytochrome c can neutralize free radicals but can not prevent cellular damage by ROS [38, 72]. Increased ^.^OH generation may occur as an end result of either enhanced peroxidase activity or decreased Cu-binding affinity of mtSOD1. mtSOD1 transgenic (Tg) mice show elevated levels of protein and lipid oxidation at both pre- and post-symptomatic stages of MN dysfunction [74, 75]. Oxidative stress is also associated with many other proposed disease mechanisms such as excitotoxicity and axonal transport defects [76–80]. Studies suggest that ROS generated in MNs can cross the plasma membrane to produce oxidative damage to glutamate transporters in neighboring astrocytes via the excitotoxic stimulation of AMPA/kainate receptors, followed by locally restricted excitotoxicity. This initiates a vicious cycle of MN overactivation causing damage, which in turn further activates MNs [37, 46, 62].

**Figure 1:**
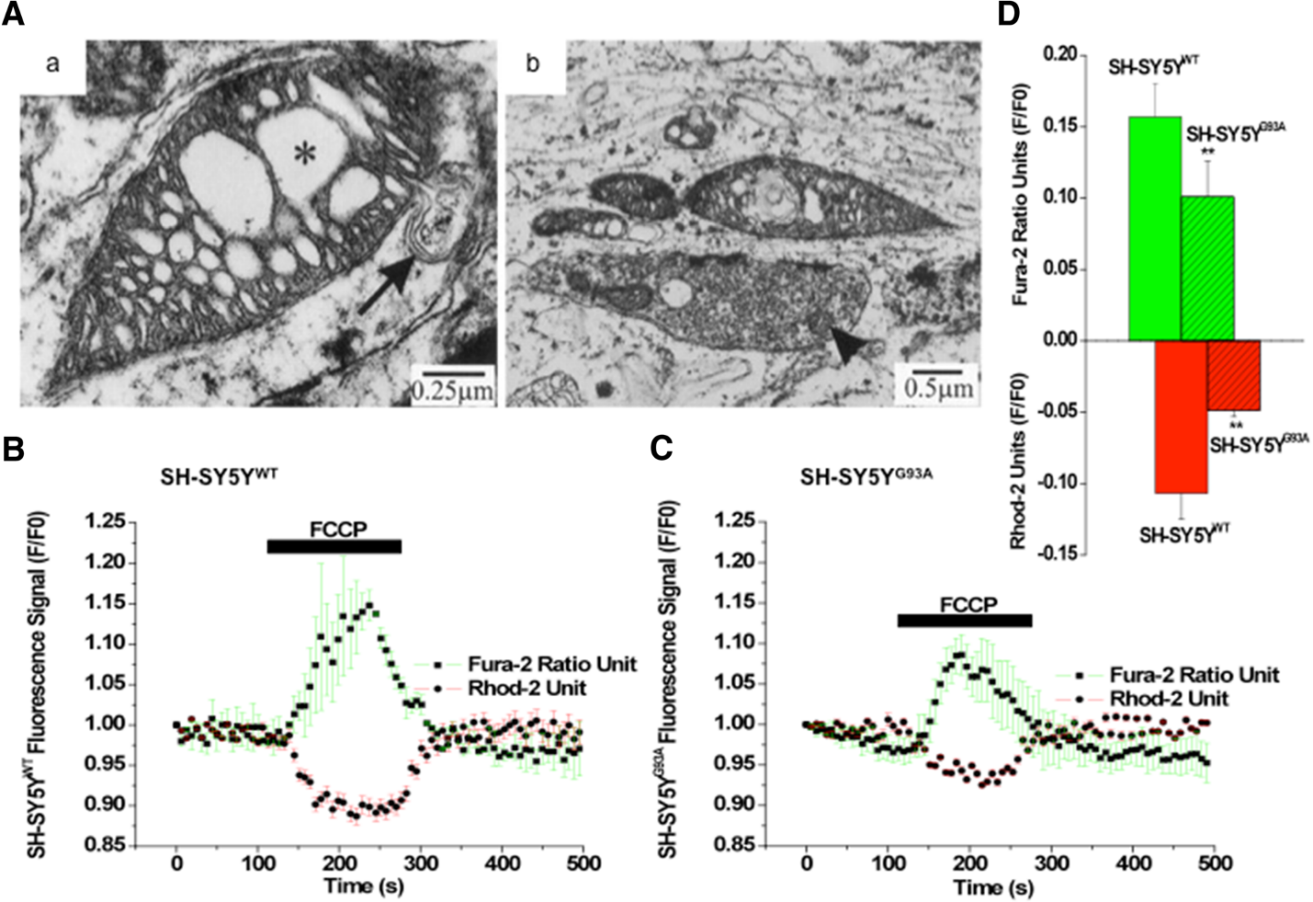
**Mitochondrial structure of motor neurons in mutant SOD1 transgenic mice and calcium load in microdomains in a cell culture model of motoneuron disease. (A)** a, Shows abnormalities like dilated cristae (asterisk) and leaking outer membrane (indicated with arrow) in mitochondrion. **(A)** b, Swollen dendritic mitochondria with dilated and disorganized cristae (adapted from ref. 31). **(B-D)** The simultaneous measurement of cytosolic calcium (Fura-2) and mitochondrial calcium (Rhod-2) concentrations in WT and G93A transfected SH-SY5Y cells during FCCP-evoked mitochondrial Ca^2+^ release. **(B)** The kinetic profile of the FCCP-evoked Ca^2+^ release in the WT transfected SH-SY5Y neuroblastoma cells; the cytosolic (Error bar green, black square trace) and mitochondrial (Error bar red, black circle trace) compartment were measured simultaneously. The trace represents the mean of 5 cells in focus stimulated with 2 μM FCCP. **(C)** The corresponding kinetic profile of the FCCP-evoked Ca^2+^ release in the G93A transfected SH-SY5Y neuroblastoma cells; the cytosolic (Error bar green, black square trace) and mitochondrial (Error bar red, black circle trace) compartment were measured simultaneously. The trace represents the mean of 5 cells in focus stimulated with 2 μM FCCP. FCCP-evoked [Ca^2+^]mito signals were smaller in amplitude and exhibited slower kinetics in G93A transfected SH-SY5Y cells compared to WT transfected cells and were altered from [Ca^2+^]i efflux. **(D)** A bar diagram of the cytosolic (green bar) and mitochondrial (red bar) fluorescence signals (F/F0) from WT (F/F0 = 0.1569 ± 0.0235 for [Ca^2+^]i and F/F0 = −0.1069 ± 0.0181 for [Ca^2+^] mito; hollow; N = 5, n = 17) and G93A (F/F0 = 0.1008 ± 0.0248 for [Ca^2+^]i and F/F0 = −0.0486 ± 0.0043 for[Ca^2+^]mito; striped pattern, N = 4; n = 17) transfected SH-SY5Y neuroblastoma cells. Values represent means ± SD, **p < 0.001. N = Number of experiments; n = Number of cells (adapted from ref. 94).

Morphological and ultrastructural abnormalities observed in mitochondria of both, sporadic and familial forms of ALS point towards a crucial involvement of mitochondria in ALS [81–84]. Localization and aggregates of SOD1 in mitochondria of transgenic mouse models were shown previously [85–87]. There are now several observations suggest that mitochondria play a crucial role in disturbing energy metabolism by predisposing calcium-mediated excitotoxicity, leading to ROS generation and initiation of the apoptotic pathway, thereby jeopardizing cell function and normal cellular metabolism [88, 89]. Defects in mitochondrial function have been found in fALS and some sALS by histopathological observations of mitochondrial swelling and vacuolization in ALS transgenic mouse models and in ALS patients [28, 30, 31, 39, 62, 90]; Figure 1A. Morphological abnormalities were not only confined to CNS, but were also found in skeletal muscles, intramuscular nerve fibers and proximal horns of the spinal cord [91–93]. Recently, in a SOD1-transfected cell culture model of MN disease, our laboratory has shown impairement of mitochondrial calcium handling and impaired cross-talk between mitochondria-endoplasmic reticulum (ER) microdomains [94]; Figure 1B-D. Many other studies have recently focused on mitochondrial dysfunction, specifically on the increased activity of the mitochondrial respiratory chain necessary for ATP synthesis, resulting in an increased ROS production. Furthermore, the excessive electron transport chain activity can deplete energy stores, resulting in the loss of integrity of neuronal cell membranes and leaving them permeable to ions and water which can cause damage. Deficits in the activities of complex I and complex IV, as a result of mutations in mitochondrial DNA, have been identified in the skeletal muscles and spinal cord of sALS patients [33, 95, 96].

The query of whether changes in the mitochondrial genome can cause variations in mitochondrial function has been addressed by transferring mitochondrial DNA from ALS subjects to mitochondrial DNA-depleted human neuroblastoma cells. This manipulation resulted in anomalous electron transport chain function, increases in the activity of free radical scavenging enzymes, disturbed Ca^2+^ homeostasis and altered mitochondrial structure, suggesting a pathophysiological role for mitochondrial DNA mutations in different forms of ALS [94, 97]. A striking recent set of publications provides evidence that mtSOD1 might disrupt the association of respiratory complex IV (cytochrome c) with the inner mitochondrial membrane, thus obstructing the mitochondrial respiratory system. Cultured MNs expressing mtSOD1 and MNs in *ex-vivo* brain slices where respiratory chain complex IV was blocked by cyanide or azide also shows mitochondrial involvement [98–100].

The major question of whether mitochondrial anomalies are involved in the disease progression or simply a derivative of neuronal degeneration is still far from over. Pathological features like the occurrence of membrane-bound vacuoles in MNs in Tg mice expressing G93A or G37R suggest that mitochondrial alterations are an early consequence eliciting the beginning of the disease, instead of merely a derivative of neuronal degeneration [101, 102]. Mitochondrial vacuolization occurs by detachment of the outer membrane from the inner membrane and increase of the intermembrane space, confirmed by biomarkers studies for mitochondrial compartments. After membrane expansion, mature vacuoles form which leads to the inner membrane disintegrations [103, 104]. A recent publication demonstrates the localization of a significant fraction of SOD1 in intermitochondrial space thereby causing toxicity. Inhibition of mitochondrial respiratory metabolism is reported in Tg ALS mice models [105, 106]. Certainly, MNs are highly susceptible to mitochondrial damage. Studies using mitochondrial respiratory chain inhibition by cyanide and azide result in selective MN death, which can be counteracted by ROS scavengers and AMPA_R_ blockers [62, 107]. Furthermore, ALS-like symptoms can be induced by deletion of vascular endothelial-cell growth factor (VEGF) that eliminates the ability to respond to tissue mild and chronic hypoxia [108–110]. Cross-breeding these mice with the mtSOD1 severely enhanced MN degeneration, while treatment of SOD1-Tg mice with VEGF hindered progression of disease symptoms and extend mice survival [62, 111–117].

### Characteristically low Ca^2+^ buffering capacity of motoneurons and its impact on selective motoneuron vulnerability in amyotrophic lateral sclerosis

**Figure 2:**
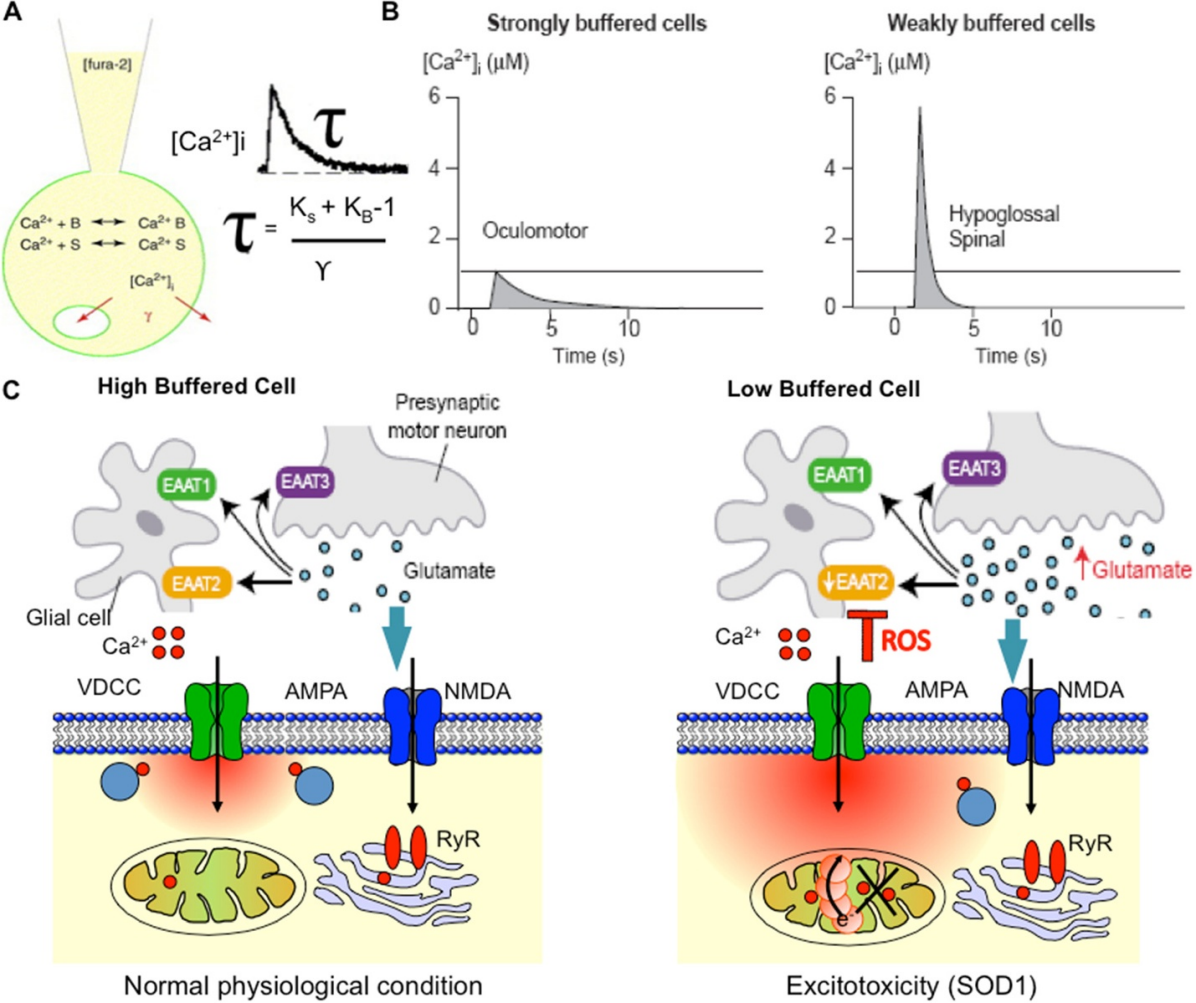
**Ca** ^**2+**^ **homeostasis and its correlation with weakly and strongly buffered motoneurons under physiological and pathophysiological conditions. (A)** The Ca^2+^ buffering capacity (K_S_) of a cell, reflecting relative fraction of bound versus free Ca^2+^, can be calculated by using the ‘added buffer’ approach by linear one-compartment model. The recovery time of [Ca^2+^]_i_ elevations (τ) depends on the amount of endogenous buffer (S; denotes Ca^2+^-binding proteins), the amount of exogenous buffer (B; i.e. Fura-2) and the transport rate (γ) of Ca^2+^ across cellular membranes. K_B_ indicates the buffer capacity of the exogenous buffer (i.e. Fura-2). **(B)** Ca^2+^ homeostasis in weakly and strongly buffered MNs. The amplitude of Ca^2+^ transients is several times larger in weakly buffered cells (e.g. HMNS and SMNs) than in strongly buffered cells (e.g. oculomotor neurons), and the recovery time is significantly accelerated (τ). **(C)** Low Ca^2+^ buffering in ALS-vulnerable HMNs exposes mitochondria to higher Ca^2+^ loads compared to high-buffered cells. Under normal physiological conditions the neurotransmitter opens glutamate, NMDA and AMPA receptor channels along with VDCC with high glutamate release and reuptake by EAAT1 and EAAT2. This results in a small rise in intracellular calcium that can be buffered by the cell. In ALS disorder, the glutamate receptor channels possess high calcium conductivity and thereby high Ca^2+^ loads; increase the risk for mitochondrial damage. This triggers mitochondrial production of reactive oxygen species (ROS), which then inhibit glial EAAT2 function. This leads to further increase in glutamate concentrations in the synapse and further rises in postsynaptic calcium levels which contributes to the selective vulnerability of MNs in ALS. Low cytosolic Ca^2+^ buffering capacity promotes Ca^2+^ accumulation and formation of subcellular domains around influx sites (red), and thus facilitates the interaction of elevated calcium levels with intracellular organelles such as mitochondria (modified from refs. [62, 63, 73, 94, 118]).

Several groups have reported that, the disruption of intracellular Ca^2+^ homeostasis plays a prominent role in the etiology of ALS. The involvement of Ca^2+^ as a risk factor was suggested by the observation that Ca^2+^-binding proteins such as CB-D_28k_ and PV were absent in MN populations lost early in ALS. In contrast, MNs less prone to damage expressed markedly higher levels of calcium-binding proteins CB-D_28k_ and/or PV [22, 62], and were relatively insensitive to mitochondrial calcium buffering. In dorsal vagal neurons, which contain an abundance of Ca^2+^ sequestering proteins [118], the delay in the decay time constant (τ) of Ca^2+^ transients (FCCP influx) is not caused by mitochondrial permeability. This observation identified a low cytosolic Ca^2+^ buffering capacity as an important risk factor for MN degeneration. Data from different groups shows that the vulnerable populations of MNs display low endogenous calcium buffering capacity [119], due to low expression levels of Ca^2+^-buffering proteins. Although potentially essential under physiological conditions, as it allows for rapid Ca^2+^ transients relaxation times during high frequency rhythmic activity, these characteristics make MNs more susceptible to an excessive influx of Ca^2+^ ions. This susceptibility increases the risk of activation of excitotoxic second messenger cascades and related cellular damages [62, 119]. Another argument in favor of this hypothesis is that high concentrations of mobile buffers accelerate the distribution of local Ca^2+^ gradients by a mechanism known as buffering diffusion (Figure 2A, B). According to this concept, under pathophysiological conditions, differential buffering reflects a basic diversity in the spatio-temporal organization of Ca^2+^ signaling rather than a singular difference in single cellular parameter [120–122]. Likewise, an increase in [Ca^2+^]i buffering capacity could defend vulnerable MNs and protect from degeneration both *in vitro* and *in-vivo* [25, 123].

In order to check whether cytosolic [Ca^2+^]i buffering can protect cells from dysfunction and degeneration, our group performed Ca^2+^ imaging studies following a depolarization stimulus (60 mM K^+^) in primary neuronal cells obtained from mice cortex at E_18_ expressing low and high CB-D_28k_. Results show that indeed CB-D_28k_ buffer [Ca^2+^]i where low CB-D_28k_ transfected neuronal cells display a significant (~2 times) reduction in the peak amplitude of the sustained [Ca^2+^]i increase compare to high CB-D_28k_ transfected neuronal cells (data not shown). *τ* in CB-D_28k_ transfected cells is also slower (~60s) compared to non-transfected cells where baseline recovery time is ~30-35 s while showing little differences in the area under the time - concentration curve (AUC). This observation is in good agreement with the ∁low buffering hypothesis” which states that low buffer capacity allows for rapid Ca^2+^ dynamics during physiological activity, but represents a significant risk factor during ALS-related MN disease [124]. The observation of high cytosolic buffering capacity in selectively resistant MNs is consistent with earlier immunocytochemical studies of endogenous calcium buffering proteins. Moreover, *in-vitro* cell culture models have shown that elevated [Ca^2+^]i buffer concentration reduces ALS specific MN damage providing further support in favor of the notion that increased buffer concentrations create beneficial protection [125]. The low Ca^2+^ buffering properties synergize with a high AMPA/kainate current density to clarify the susceptibility of MNs to increased stimulation by glutamate and associated Ca^2+^ influx [59, 126]. Earlier observation also indicates interactions of mitochondrial proteins e.g. VAPBP56S and PTPIP51 implicated in regulating calcium homestasis [127, 128].

In the absence of adequate cytoplasmic buffering, or in cases where the existing cytoplasmic buffering system is overwhelmed, there is a shift in mitochondrial Ca^2+^ uptake from a beneficial physiological regulatory mechanism to a possibly detrimental process leading towards MNs death. In this process MNs death is clearly Ca^2+^ dependent. The downstream mechanism that links the rise in [Ca^2+^]_i_ to MNs death is not clear though these processes have been mostly credited to mitochondrial Ca^2+^ accumulation or glutamate excitotoxicity. Whether the change in ΔΨ_m_ is simply an inevitable consequence of an unusually enormous Ca^2+^ load or there is other downstream pathway contributing to glutamate response is not known yet, however depolarisation of ΔΨ_m_ is one consequences of it. Data also suggested the role for other factor in addition to Ca^2+^ such as nitric oxide synthase (NOS) inhibitors. Results also suggest that mitochondrial damage arbitrated at least in part by Ca^2+^ induced MPTP opening and may contribute to surge of neighbouring cell activation. In conclusion, we believe that two of the most important features for MNs in ALS are: (i) low buffer capacity generates exceptionally large Ca^2+^ domains, but not in case of serious end stage ALS like symptoms and (ii) in ALS vulnerable MNs buffering capacity critically depends upon the domain size of mitochondria and ER. Therefore, we proposed a model where a portion of MN mitochondria interacts with areas of high [Ca^2+^] around influx sites due to low buffering induced excitotoxicity shown in Figure 2C.

Another proposed factor for calcium disregulation could be an ALS-related immune response targeted at voltage-dependent calcium channels (VDCC), where a disruption of Ca^2+^ homeostasis results from compromised voltage-dependent calcium influx [129]. Furthermore, synaptic glutamate transport is also believed to be involved in other forms of ALS and related MN neurodegeneration. In cell-culture, limited protection was achieved by treatment with nifedipine, implicating Ca^2+^ entry through voltage-gated Ca^2+^ channels, in addition to glutamate receptors, in mediating the toxicity of mtSOD1 in MNs. The crucial role of Ca^2+^-permeable AMPA_R_ was further emphasized by cross-breeding of Tg SOD1 mice with mice that exhibited markedly reduced Ca^2+^ permeability of AMPA/kainate receptors, due to GluR2 overexpression [45, 126]. Finally, impaired mitochondrial calcium transport capacity in mtSOD1 mice may play an important role. Firstly, it links mitochondrial dysfunction to glutamate excitotoxicity and secondly, elevation of [Ca^2+^]_c_ concentrations in neurons compromises mitochondrial integrity and function by inducing enhanced production of ROS from mitochondria [130]; Figure 2C.

### Glutamate transmission and excitotoxicity

Glutamate is known as the predominant excitatory neurotransmitter in the CNS acting at both ionotropic and metabotropic receptors. It is synthesized and stowed in synaptic nerve components and released in response to depolarization of the neuron. Excessive glutamate exposure is toxic to neurons via glutamate-triggered Ca^2+^ influx [73, 131]. Several lines of proof implicate increases in glutamate neurotransmission and glutamate-triggered Ca^2+^ entry as significant ALS risk-factors. Increased extracellular glutamate levels result from reduced glial glutamate uptake due to oxidative damage to excitatory amino acid transporter 2 (EAAT2) or by aberrations in its production. Increased glutamate levels in the cerebrospinal fluid (CSF) of a subset of ALS patients have been shown by many groups. The elevation of this glutamate level may be attributed to deficient glutamate transporter capacity (loss of EAAT2 function), as low levels of the transport protein have been found in some post mortem ALS brains [66, 73, 132]. Furthermore, glutamate uptake inhibitors causes selective MN damage in organotypic slices [66] and in dissociated spinal cord culture [133], suggesting that reduction of glutamate transport could contribute to the MN damage seen in the ALS disease. The leading argument for a role of glutamate excitotoxicity in ALS is the efficacy of FDA approved drug riluzole, the lone drug which proved effective against disease progression in patients and has anti-excitotoxic properties. It was shown that riluzole inhibits the release of glutamate through the inactivation of voltage-dependent Na^+^ channels on glutamatergic nerve terminals as well as to activate a G-protein-dependent signal transduction cascades.

*In-vivo* evidence for a possible role of GluR1-4 (AMPA receptor subunits) in ALS comes from several studies. Transgenic mice lacking GluR2 (GluR2 subunit is a component in the AMPA receptor complex, which renders them particularly impermeable to calcium) do not hurt from MN disease. This suggests that a low GluR2 level is a modifier of MN degeneration rather than being sufficient to cause ALS [134]. Furthermore, glutamate excitotoxicity in sALS is caused by a selective loss of astrocytic glutamate transporter-1 (GLT-1) and is reproduced in mice by knockout of GLT-1, a homologue of EAAT-2 [135]. Oral administration of glutamate inhibitors prolonged the life span of SOD1^G93A^ mice [136]. Further studies have pointed to the significance of GluR2 in neuronal survival, in which alterations in RNA editing at the Q/R site lead to the generation of a lethal phenotype involving seizure and acute neurodegeneration [137]. Furthermore, Glu2-N overexpression induces a progressive decline in the function of spinal cord, most likely due to the long-onset degeneration of spinal MNs [138].

### Mechanism underlying mitochondria-ER Ca^2+^ stores coupling

Diverse microdoamin intracellular pools contribute in causing Ca^2+^ signals in neuronal cells and in shaping their spatio-temporal patterns and cell fate. Kinetic and “hot spot” hypothesis of mitochondria, different channels with distinct properties and highly defined expression patterns on ER are all capable of regulating [Ca^2+^]_i_ in many systems [139, 140]. In an attempt to understand more about the Ca^2+^ metabolism of hypoglossal MNs, Jaiswal and colleagues studied the role of the ER in Ca^2+^ handling where it was shown that the ER in MNs retained a comparatively lower quantity of calcium than mitochondria after [Ca^2+^]_i_ elevation, indicating a relative inability to sequester Ca^2+^ in the MNs of SOD1^G93A^ mice as compared to WT lettermates. These results indicate that the conventional Ca^2+^ storing function of mitochondria is dominating over ER Ca^2+^ accumulation in these MNs. These results are in good agreement with the “hotspot” hypothesis that suggests that mitochondria preferentially accumulate Ca^2+^ at microdomains of elevated Ca^2+^concentration ([Ca^2+^]i), predominantly near ER Ca^2+^ release sites and other Ca^2+^ channels. Accordingly, mitochondria can affect both Ca^2+^ release from the ER and capacitative Ca^2+^ entry across the plasma membrane, thereby shaping the size and duration of the intracellular Ca^2+^ signal in MNs of WT and SOD1^G93A^ mice. These events determined by the Ca^2+^ sensitivity of the Ca^2+^ channels and capability of mitochondria to remove Ca^2+^ from the subcellular microdomain at the opening of the ion channel. This effect has been confirmed *in-vitro*, but the condition appears markedly different in various cell models [141, 142]. This indicates that several modulatory mechanisms occur, many of which still await reasonable clarification at cellular and molecular level. As discussed above, vast evidence supports the notion that the measured high degree of Ca^2+^ accumulation of MNs mitochondria *in-situ* mainly influenced by the vicinity of mitochondria to the ion channels through which Ca^2+^ enters the cytosol. A fundamental, but still unanswered, question is the precise mechanism by which stochastic versus specific localization of Ca^2+^ influx occur and the amount to which mitochondrial function differs within different cell types, are critical quest in the field of ALS research.

### Mitochondrial dysfunction, Ca^2+^ homeostasis and ALS: a multifactorial disease mechanism

The Ca^2+^-dependent signaling mechanisms that result in the enhanced vulnerability of MNs in ALS disease and associated mouse models are also critical for normal cellular function. Earlier studies suggest that unrestrained Ca^2+^ entry compounded with an inability to sequester this calcium leads to the degeneration of mitochondria in the MNs of the mouse model of ALS [29, 31]. In other MNs types have a low Ca^2+^-buffering capacity because of small concentrations of Ca^2+^-buffering proteins and a high quantity of Ca^2+^-permeable AMPA_R_ [126, 143–146]. These two properties appear to be specific to MNs and are possibly vital for their normal function. However, this increased sensitivity to calcium also means that MNs are more easily over stimulated by glutamate and astounded by Ca^2+^. It is unknown whether Ca^2+^ dependent pathways are different in MNs. More recently, focus was shifted to the role of mitochondria as an effective regulator of [Ca^2+^]i signals [147, 148]. The use of mitochondria-targeted Ca^2+^ probes reveals a fast, intense surge in free intra-mitochondrial Ca^2+^ upon cellular stimulation. Increased Ca^2+^ uptake by mitochondria leads to up-regulation of the enzymes activity in oxidative metabolism, resulting in cell-specific metabolic changes [149–151]. This hypothesis is further strengthened by the appearance of abnormalities in mitochondrial ultrastructure and vacuoles formation resulted from degenerating mitochondria found in post mortem samples of ALS [28–31]. Even though the precise molecular mechanism is still not known, we hypothesize that MN vulnerability in ALS is a outcome of physiological features, mainly highly specialized Ca^2+^ surroundings that are required for appropriate neuronal function, uninterrupted activity-dependent mitochondria-ER Ca^2+^ cycling and the leading role of mitochondria in buffering Ca^2+^ transients.

In vulnerable MNs, low instrinsic cytosolic Ca^2+^ buffering requires that mitochondria play the dominant role in the regulation of [Ca^2+^]_i_ transients; even during small cytosolic Ca^2+^ increases, mitochondria are known to take up more than 50% of intracellular Ca^2+^. Outsized and enduring Ca^2+^ microdomains around Ca^2+^ influx sites increase the danger of toxic Ca^2+^ buildups and a successive activation of Ca^2+^ dependent neurodegenerative pathways under excitotoxic conditions. Excitotoxicity associated with these influx domains is primarily suppressed by mitochondrial contributions, rather than ER Ca^2+^ uptake [36, 94, 99]. The prominent role of mitochondria in regulating adequate Ca^2+^ loads in MNs has significant implications for pathological conditions such as in ALS. First, the quantity of Ca^2+^ taken up by the mitochondria is greater in MNs than in many others cell types and therefore MN mitochondria are more susceptible to Ca^2+^ mediated damage, such as ROS generation [37, 46, 152]. Second, our experiments provide indication that the restriction of cytosolic Ca^2+^ to non-pathoological levels is determined by intact Ca^2+^ uptake into mitochondria. Hence, when mitochondrial Ca^2+^ uptake is disturbed as seen by low Ca^2+^ uptake in SOD1^G93A^ MNs compare to WT, MNs are directly endangered by elevated Ca^2+^ levels, especially during high-frequency, repetitive Ca^2+^ oscillations. The reduced capability to limit Ca^2+^ transient amplitudes in the cytosol, particularly in local microdomains of extraordinary Ca^2+^ influx when mitochondria are depolarised, heightens the hazard of initiating Ca^2+^ dependent neurodegenerative pathways leading to cell demise.

Combining the lessons learned from multiple animal and cell culture models of ALS, the central insight is that mitochondrial dysfunction and Ca^2+^ homeostasis [152, 153] are strong contributors to the selective vulnerability of MNs. Considering the association and significance of mitochondrial dysfunction and Ca^2+^ homeostasis, we postulate that MN possess large number of voltage and ligand gated Ca^2+^ channels that, when activated, cause rapid Ca^2+^ influx. Since cytoplasmic Ca^2+^ buffering is relatively weak in these cell types, significant demand is placed on MN mitochondria. If the mitochondria are already damaged or weakened, this results in mitochondrial Ca^2+^ overload and ROS production and in some cases prone for infection [154]. Furthermore, long-lasting ΔΨ_m_ depolarisation due to Ca^2+^ entry can be a basis for the release of pro-apoptotic proteins and activate enzymes involved in apoptotic pathways [155, 156].

### Multidrug therapies in ALS: where should we focus for the treatment?

Even though scientific discoveries are speeding up with an exceptional pace and years of experimentation using cell cultures, mice and rat made Tg for human mtSOD1 has yielded precious data about the mechanisms that underlie ALS as well as suggestions for therapy, to date approaximately ~50 clinical trials have failed and ended with disappointment and frustration. The main cause for the failure to translate experimentation in animals to therapies for patients is that there are too many compounds that increase life span in mice and rats, but fail to improve human patients condition. To increase the chances of success for future clinical trials we might consider the fact that ALS is not simply a multifactorial disease but also a multisystemic disease that is the consequence of a complex neurotoxic mechanism that involves molecular and cellular cross-talk between MNs, glia and astrocytes. Prelinical trials have not yet resulted in favorable outcomes, due to the fact that data are generally collected in animals of the identical age and with the same mutation expressed in a homogeneous genetic background whereas the age of beginning of disease, the progression and the severity of ALS in human patients are heterogeneous, signifying that possible genetic risk factors and modifying causes exist for sALS and certain drugs are effective only if given before onset of the disease [157–159]. In addition, the trial suffers from the deficiency of presumed negative clinical data set, which is different from the null result (i.e. data that do not affect the outcome). Interestingly, in most of the clinical trials, a subset of the patient population showed better condition with the possibility that each clinical trial has been successful within only a select subset of the patient population [158].

It is also important to keep in mind that ALS is a multifactorial disease, and it might be unrealistic to envision that one drug will have a broad spectrum of efficacy on pathologies that are widespread and, at times unrelated. In view of recent findings of a non-cell-autonomous demise of MNs [47, 48], design of multi drug combination therapies should be targeted at the intersection of various aspects of this cascade, rather than a single-drug cure [157, 158]. We suggest that the forthcoming clinical trials should include combinatorial studies, and that patients who display progress in their condition should not be considered as ‘outliers’, because they might indeed represent the target population, especially for the drugs tested [157]. Until now animal studies have shown that multi-drug combination therapies and the method of delivery of a drug is also often have synergistic effects in ALS, for example, riluzole administered with melatonin and vitamine E (inhibits Na^+^-current activation and the apoptotic cascade), minocycline administered with creatine (inhibits microglia activation and the apoptotic signaling cascade), or treatment with IGF-1 or VEGF retrogradely transported in MNs through viral vectors [111–119, 136, 158–163]. A substitute to pharmacological cures, the latest developments in stem-cell therapy might offer possibilities for neural grafting in patients with ALS [114, 158, 164]. The identification and characterization of early detection markers in ALS, and the establishment of dependable biomarkers for disease progression in a select set of clinical trials studies on blood, plasma and cerebrospinal fluid (CSF), obtained from patients and control subjects bounds to improved improved clinical trials [165]. The lessons learned from a decade of research using the mtSOD1 animal model might help scientists in finding cures for neurodegeneration where single-drug treatments have confirmed insufficient for effective treatment of ALS.

## Conclusions

In spite of rigourous research for years there are several important questions still unanswered; these include: (a) does the expression of mtSOD1 at physiological levels causes morphological and structural abnormalities of mitochondrial assembly and its calcium buffering capacity? (b) At pathophysiological levels, does the mitochondria-ER and EMRCC Ca^2+^ sequestration source specificity and spatiotemporal properties of [Ca^2+^]i signaling varies at sub cellular level in and around microdomain? (c) In the presence of mtSOD1 gene, what are the consequences of alterations in mitochondrial function on Ca^2+^ homeostasis and ERMCC? Numerous developments and improvements in visualization of diseased MNs and spatiotemporal resolution of mitochondria-ER calcium signaling cascades have the potential to bring novel insights. MN possess numerous Ca^2+^ channels that cause rapid Ca^2+^ influx because of comparatively weak [Ca^2+^]i buffering, results in mitochondrial Ca^2+^ overload and strong ROS generation in mtSOD1 Mice. Further studies of ER-mitochondria calcium cycle (ERMCC) with targeted calcium probes show a rapid and dramatic increase in free intra mitochondrial and ER calcium. The defects in mitochondrial assembly and vacuoles derived from defective mitochondria found in post mortem ALS patients further support the suggestion. Design of novel antioxidant strategies to selectively target the oxidative stress and redox imbalance is the other evenue need to be explored. Furthermore, check on selective loss of MNs causing the discharge of pro-apoptotic proteins which activate enzymes leads to activation of apoptotic pathways and apoptotic cells observed in MNs of ALS is beneficial. Dysregulation between mitochondria-ER and ERMCC are well known features and therapeutic drugs aiming to stabilize these cycles reduce ROS and oxidative stress and may be effective in wide range of MN diseases. Another nice direction would be to develop so called “smart drugs” and “combination therapies or multi drug therapies” which have mutifactorial imact on disease mechanism due to mulfactorial nature of ALS disease. Furthermore, it is hypothesized that disrupted Ca^2+^ homeostasis and oxidative stress induced ROS have a vital role in propagating injury by increasing the excitability of MNs and by targeting neighboring glia. Perhaps as a consequence, excitotoxicty builds up with increased activity-dependent Ca^2+^ influx and associated mitochondrial Ca^2+^ cycling. Given the mitochondrial disturbances, Ca^2+^ buffering becomes inefficient and increase in cytosolic Ca^2+^ levels. Protective options are to elevate the resistance of MNs to high intracellular Ca^2+^ concentrations by inducing defense mechanism and/or to inhibit the downstream apoptotic and death cycle pathways activated by increased intracellular Ca^2+^ concentrations. However, severely impaired MNs are not amendable to taking functional advantage of neuronal protection in ALS.

Therefore perhaps we should focus on new tools such as recently discovered genes that cause ALS and induced pluripotent stem cells taken from ALS patients and derived into MNs to identify potential cytosolic pathways and barriers that could lead to MN degeneration in ALS. Forthcoming studies will hopefully add to the understanding of why these processes preferentially damage MNs and the role non-cell autonomous cell death might play. In conclusion, data indicates that ALS is a multifactorial disease and therefore a combined therapeutic interference (combination therapy) with many facets of target site both at the MNs and glial cells will be most likely essential for survival of ALS patients. While keeping in mind the previous failures in clinical trials for ALS, further studies in this direction to better understand the pathogenesis of cell death in ALS and targeted therapy therefore will be of great interest.

## Abbreviations

ALS: Amyotrophic lateral sclerosis
aCSF: Artificial cerebrospinal fluid
AMPA: Alpha-amino-3-hydroxy-5-methyl-4-isoxazolepropionic acid
[Ca^2+^]i: AMPA_R_, AMPA receptors
[Ca^2+^]_c_: Cytosolic calcium
CB-D_28K_: Calbidin D_28K_
CSF: Cerebrospinal fluid
EAAT2: Excitatory amino acid transporter 2
ER: Endoplasmic reticulum
ERMCC: ER-mitochondria calcium cycle
fALS: Familial amyotrophic lateral sclerosis
FCCP: Carbonyl cyanide 4-(trifluoromethoxy) phenylhydrazone
FMNs: Facial motoneurons
GluR: Glutamate receptors
GLT: Glutamate transporter
hALS: Human amyotrophic lateral sclerosis
HMNs: Hypoglossal motoneurons
PV: Parvalbumin
ROS: Reactive oxygen species
SOD1: Cu/Zn superoxide dismutase 1
mtSOD1: Mutant superoxide dismutase1
MNs: Motoneurons
NMDA: N-Methyl-D-aspartic acid
PV: Parvalbumin
Tg: Transgenic
VDCC: Voltage-dependent calcium channels
VEGF: Vascular endothelial-cell growth factor
WT: Wild-type
Δψ_m_: Mitochondrial membrane potential
τ: Decay time constant.
